# Clinical observation of tongue coating of perioperative patients: factors related to the number of bacteria on the tongue before and after surgery

**DOI:** 10.1186/s12903-018-0689-x

**Published:** 2018-12-20

**Authors:** Madoka Funahara, Souichi Yanamoto, Sakiko Soutome, Saki Hayashida, Masahiro Umeda

**Affiliations:** 1 Kyushu Dental University School of Oral Health Sciences, 2-6-1 Manazuru, Kokura-kita, Kitakyushu, Fukuoka, 803-8580 Japan; 2 Department of Clinical Oral Oncology, Nagasaki University Graduate School of Biomedical Sciences, 1-7-1 Sakamoto, Nagasaki, 852-8588 Japan; 3 Perioperative Oral Management Center, Nagasaki University Hospital, 1-7-1 Sakamoto, Nagasaki, 852-8588 Japan

**Keywords:** Tongue coating, Surgery, Bacteria, Perioperative oral management

## Abstract

**Background:**

Increased amount of tongue coating has been reported to be associated with increased bacteria count in the saliva and aspiration pneumonia in elderly people. However, the implications of tongue coating for prevention of postoperative complications in patients undergoing major oncologic or cardiac surgery has not been well documented. The purpose of this study is to investigate the number of bacteria on the tongue before and after surgery and factors affecting it.

**Methods:**

Fifty-four patients who underwent oncologic or cardiac surgery under general anesthesia at Nagasaki University Hospital were enrolled in the study. Various demographic, tumor-related, treatment-related factors, and the number of bacteria on the tongue and in the saliva were examined, and the relationship among them was analyzed by Mann-Whitney U test, Spearman rank correlation coefficient, or multiple regression.

**Results:**

Before surgery, no significant factors were correlated with the number of bacteria on the tongue, and there were no relationship between bacteria count on the tongue and that in the saliva. On the next day after surgery, bacteria on the tongue increased, and sex, periodontal pocket depth, feeding condition, dental plaque, blood loss, and bacteria in the saliva were correlated with bacteria on the tongue by a univariate analysis. A multivariate analysis showed that feeding condition, and amount of dental plaque were correlated with the number of bacteria.

**Conclusions:**

Increased number of bacteria on the tongue was associated with feeding condition and amount of dental plaque. Further studies are necessary to clarify the clinical significance of dental coating in perioperative oral management of patients undergoing oncologic or cardiac surgery.

## Background

Tongue coating is characterized by a white, yellowish brown, or black mossy coating on the surface of the tongue. Tongue coating is formed by hyper-keratinization and elongation of the tongue papillae on the dorsal surface of the tongue, and the presence of oral bacteria, exfoliated epithelium, or food residue among the papillae. When these bacteria produce pigments, the tongue coating may appear yellowish brown or black. An abnormal quantity and quality of tongue coating are thought to be related to dry mouth, depression of immunity, oral breathing, poor oral hygiene, smoking, old age, psychological stress, general disease, or medication side effects, etc. [1].

Cancer patients who undergo surgery, radiotherapy, or chemotherapy sometimes develop various infections including surgical site infection (SSI) or aspiration pneumonia, and those who undergo cardiac surgery may rarely develop infective endocarditis (IE). Some of these complications are thought to be due to oral bacteria, and therefore, oral health care is recently recognized to be important during cancer therapy or cardiac surgery [2–4]. It has been reported that increased amount of tongue coating is associated with increased bacteria in the saliva and aspiration pneumonia in elderly people [5, 6]. However, the implications of tongue coating and the necessity of removing it in cancer patients during treatment have not been well documented. As a first step to respond to these questions and to establish standardization of oral care for perioperative patients, we decided to investigate whether bacteria on the tongue increased after major oncologic or cardiac surgery and what factors influenced the number of bacteria on the tongue.

## Methods

**Fig. 1:**
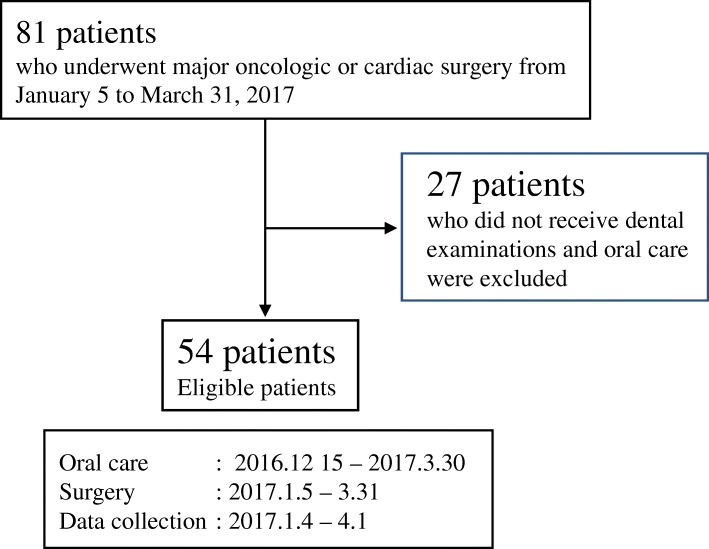
Flowchart of the study design

The study is a prospective, observational study comprising 54 patients who underwent surgery for cancer or heart disease at Nagasaki University Hospital between January and March 2017 (Fig. 1). Patients who did not perform dental examinations or oral care described below were excluded. After written informed consent was obtained from each patient, the following factors were examined: 1) patient-related factors (age, sex, body mass index, performance status [PS], diabetes, smoking habit, and drinking habit); 2) laboratory data (preoperative serum creatinine, alanine aminotransferase, and albumin); 3) treatment-related factors (indication for surgery, operation time, and blood loss during surgery); and 4) oral condition (amount of dental plaque, maximum periodontal pocket depth before surgery, feeding condition on the day after surgery, oral wetness, and number of bacteria in the saliva and on the tongue before and after surgery).

PS was categorized as PS 0 to PS 4 according to the Eastern Cooperative Oncology Group classification [7]. The amount of dental plaque was defined as the O’Leary plaque score [8] × number of the teeth. Oral wetness was measured at the surface of the buccal mucosa using an oral hydrometer (Moisture Checker Mucus®, Life Co., Ltd., Saitama, Japan). The number of bacteria on the tongue and in the saliva, was measured according to a previously reported method [9, 10] with a rapid oral bacteria quantification system (Panasonic Healthcare Co. Ltd., Osaka Japan) using the dielectrophoresis and impedance measurement methods.

Each patient received standard oral care from a dentist and dental hygienist. Oral care was started from the time the decision for hospitalization was made. It included oral health instruction, removal of dental calculus (scaling), professional mechanical tooth cleaning (PMTC), removal of tongue coating with toothbrush, cleaning denture, and extraction of tooth with severe periodontitis. All patients received final oral cleaning by a dentist or dental hygienist the day before surgery.

Statistical analyses were performed using SPSS software (version 24.0; Japan IBM Co., Tokyo, Japan). Univariate analysis of the relationship between each variable and number of bacteria on the tongue was analyzed using the Mann–Whitney U test and Spearman rank correlation coefficient. Multivariate analysis was conducted using stepwise multiple regression analysis. The differences among number of bacteria on the tongue before oral care, after oral care, and after surgery were analyzed using the Mann–Whitney U test. The correlation between number of bacteria on the tongue and that in the saliva was analyzed by the Spearman rank correlation coefficient. Probabilities of less than 0.05 were accepted as significant.

Ethical approval was obtained from the Institutional Review Boards (IRB) of Nagasaki University Hospital (No. 16031420).

## Results

### Characteristics of the patients

Table 1 shows background factors of the patients. Thirty-two patients were males and 22 females, with an average age of 64.7 years. Oncologic surgery was performed in 43 patients and cardiac surgery in 11. Ten patients fed orally at the next day of surgery, while 38 were fasted and 6 were under intubation.

**Table 1:** Background factors of the patients

Variable	Category	mean ± SD or number
Gender	male	32
	female	22
Age		67.4 ± 14.5 years
Disease	prostate cancer	1
	pancreatic cancer	1
	liver cancer	2
	kidney cancer	2
	head and neck cancer	4
	colorectal cancer	5
	breast cancer	5
	other cancers	5
	gastric cancer	7
	lung cancer	11
	heart disease	11
BMI		22.0 ± 3.33
PS	PS0	43
	PS1	7
	PS2	4
Diabetes	–	45
	+	9
Smoking habit within one year	–	48
	+	6
Drinking habit within one year	–	41
	+	13
Creatinine		0.834 ± 0.260 mg/dL
ALT		23.5 ± 18.1 IU/L
Albumin		4.05 ± 0.625 g/dL
Amount of dental plaque		492.45 ± 383.17
Periodontal pocket depth	edenturous	6
	< 6 mm	39
	≥6 mm	9
Oral wetness before surgery		26.1 ± 2.94
Oral wetness after surgery		23.7 ± 5.08
Operation time		278 ± 177 min
Blood loss		411 ± 775 g
Feeding condition on the next day after surgery	normal food	10
	stop feeding	38
	intubation	6
Number of bacteria in the saliva before surgery (logarithm)		5.54 ± 0.52
Number of bacteria in the saliva after surgery (logarithm)		6.61 ± 0.91
Number of bacteria on the tongue before surgery (logarithm)		6.75 ± 0.601
Number of bacteria on the tongue after surgery (logarithm)		6.88 ± 0.66

### Correlation between number of bacteria on the tongue and that in the saliva

**Fig. 2:**
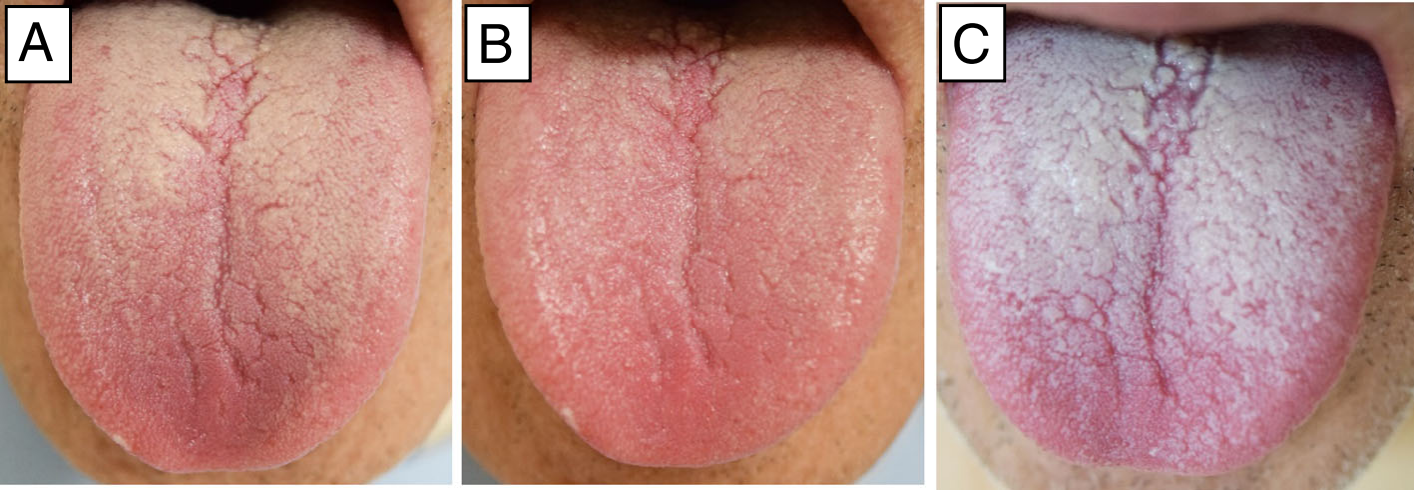
Macroscopic features of the tongue coating. **a** Before oral care, **b** After oral care, **c** After surgery

Fig. 1 shows the correlation between bacteria count on the tongue and that in the saliva before oral care on the day before surgery. No significant correlation was found between them. However, there was significant correlation in number of bacteria on the tongue and that in the saliva on the day after surgery (Fig. 2).

### Number of bacteria on the tongue and factors affecting it before and after surgery

The number of bacteria on the tongue ranged from 10^5.00^ to 10^7.89^ cfu/mL before surgery. The univariate analysis showed that no variable examined in the study was significantly correlated with the number of bacteria on the tongue before surgery (Table 2). The multivariate analysis also showed no significant factors correlated with number of bacteria on the tongue (data not shown).

**Table 2:**
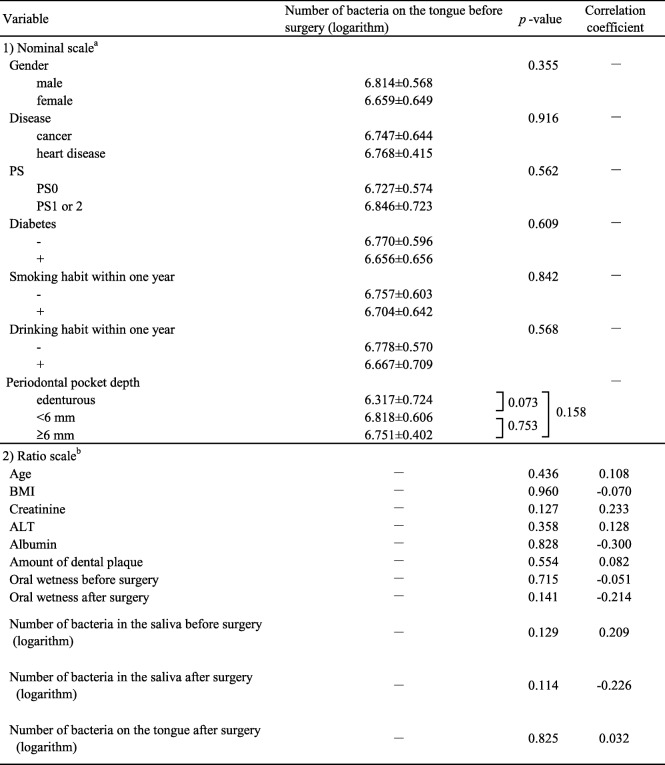
Correlation between each variable and bacteria on the tongue before surgery

^a^Mann–Whitney U-test

^b^Spearman rank correlation coefficient

On the day after surgery, most patients showed a larger number of bacteria on the tongue, ranging from 10^5.71^ to 10^8.00^ cfu/mL. The univariate analysis indicated that patient sex, periodontal pocket depth, feeding condition, and the number of bacteria on the tongue before surgery were significantly correlated with the number of bacteria on the tongue after surgery (Table 3). Multiple regression analysis showed feeding condition and amount of dental plaque to be independent significant factors related to the bacteria count on the tongue (Table 4).

**Table 3:**
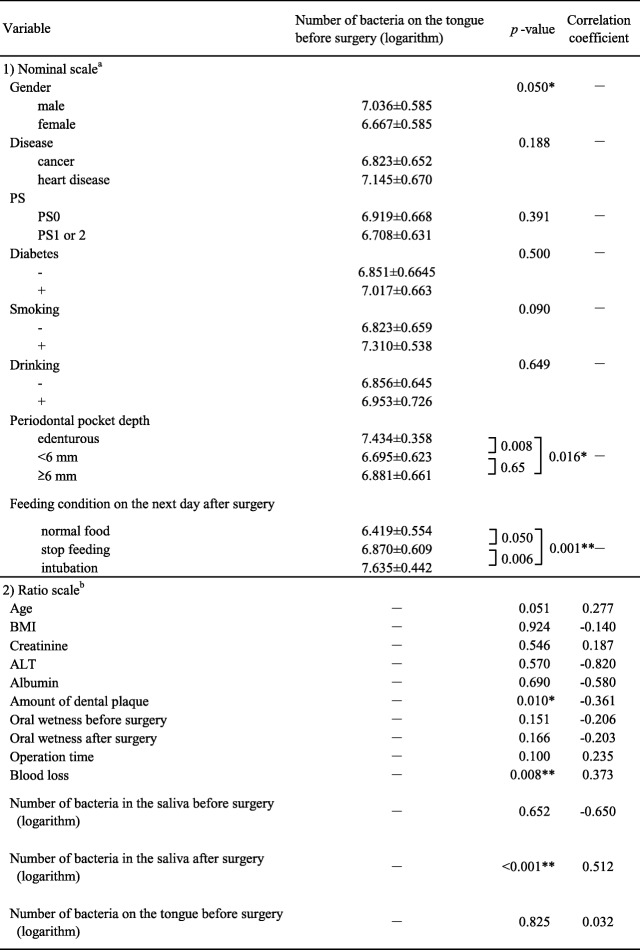
Correlation between each variable and bacteria on the tongue after surgery

^*^ means *p* < 0.05 and ^**^ means *p* < 0.01

^a^Mann–Whitney U-test

^b^Spearman rank correlation coefficient

**Table 4:** Variables with are significantly corelated with bacteria count on the tongue after surgery (Multiple regression analysis)

	B	standard devision	β	t-value	*p*-value	95% CI of B
Feeding condition	0.505	0.152	0.423	3.32	0.002	0.199–0.811
Amount of dental plaque	0.000	0.000	−0.275	−2.160	0.036	−0.001-0.000

### Perioperative changes of number of bacteria on the tongue according to feeding condition

**Fig. 3:**
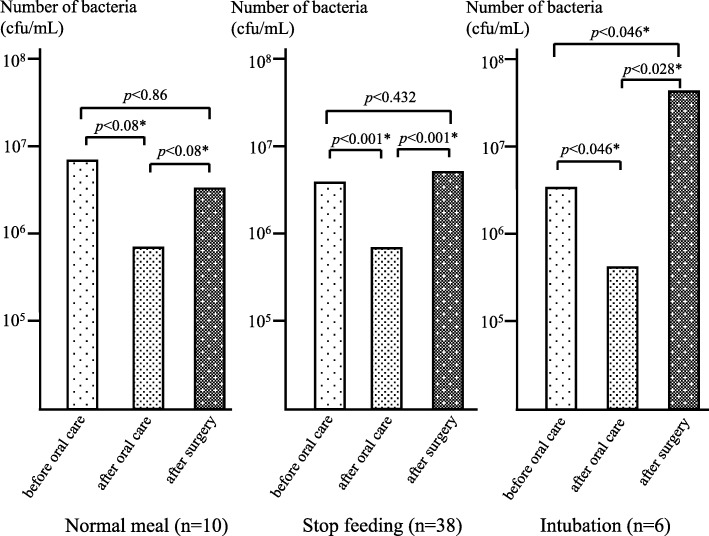
Change in bacteria count on the tongue before and after surgery (Wilcoxon rank sum test)

On macroscopic inspection, most patients showed thicker tongue coating after surgery (Fig. 3). demonstrates the number of bacteria on the tongue before and after surgery. After preoperative oral care, bacteria count was significantly reduced, but on the day after surgery, it increased again. The degree of increase was the smallest in patients who ate normal food immediately after surgery, intermediate in those who were fasting the day after surgery, and largest in intubated patients.

## Discussion

In elderly people need in care, pneumonia is mostly believed to be caused by aspiration of pathogenic microorganisms in the saliva, as well as swallowing disorder and depressed immunity [11]. Some authors indicate that bacteria in the dental plaque or periodontal pockets is a reservoir for oral bacteria and, therefore, may become one of the major causes of aspiration pneumonia [12, 13]. There are various methods for evaluating the state of oral hygiene, and we used the amount of dental plaque as the O’Leary plaque score × number of the teeth in the study. Other evaluation methods such as Oral hygiene index- debris index (OHI-DI) [14] are also useful, but since the number of samples in this study is small, we focused on quantifying detailed plaque and used the O’Leary plaque score × number of the teeth. However, occurrence rate of pneumonia did not differ between dentulous and edentulous elderly patients need in care [11], which indicates that neither dental plaque nor periodontal disease is the main cause of aspiration pneumonia in the elderly. In contrast, Abe et al. [5] reported that tongue coating is associated with number of viable salivary bacterial cells and the development of aspiration pneumonia, and that tongue coating is a risk indicator of aspiration pneumonia in edentate elderly people in nursing homes. Additionally, Ryu et al. [6] have reported a relationship between tongue coating status, as well as salivary flow rate, denture plaque, and frequency of oral self-care, and number of oral anaerobic bacteria; however, the implication of tongue coating in perioperative patients has not been well documented.

Hayashida et al. [10] reported that tongue coating in intubated patients increased immediately after surgery, although dental plaque did not. We examined the number of bacteria in various sites of the oral cavity during surgery under general anesthesia, and found that bacteria on the dorsum of the tongue rapidly increased immediately after intubation but, at surfaces of the buccal mucosa and the palate, the number of bacteria did not change during surgery [9]. These findings suggest that the dorsal tongue environment is suitable for bacterial growth, especially when self-cleaning functions are reduced due to suppression of the swallowing function.

In Japan, oral care by dentist and dental hygienist before and after majora oncologic or cardiac surgery has been covered by public medical insurance system since 2012, and it was called as “perioperative oral management”. Perioperative oral management aims to prevent various complications such as SSI, postoperative pneumonia, IE, and oral mucositis during cancer therapy or cardiac surgery by reducing oral bacteria. There are two possible mechanisms by which oral bacteria are involved in these complications: direct exposure of bacteria in the saliva and hematogenous infection to distant sites from infection lesion in the oral cavity. Therefore, it is important not only to eliminate infection foci in the oral cavity, but also to reduce the number of saliva bacteria in the perioperative period. The study suggests that tongue coating is one of the main reservoirs of bacteria in the saliva, but it has not been investigated whether the number of bacteria in the saliva will decrease if tongue coating is removed.

We undertook this study in an attempt to establish standardization of oral care for perioperative patients to prevent postoperative complications such as surgical site infection and postoperative pneumonia. Most patients underwent oral care before and after surgery, which consisted of tooth brushing, scaling, professional mechanical tooth cleaning, and gargling; removal of tongue coating is not included in the standard oral care. The current study showed that the number of bacteria on the tongue was significantly correlated with that in the saliva the day after surgery, while there was no correlation between them before surgery. The difference in the preoperative and postoperative results is thought to be due to disorder of self-cleaning functions of the oral cavity after surgery, probably because of swallowing dysfunction and postoperative fasting. These results suggest that removal of the tongue coating will lead to a decreased number of bacteria in the saliva in postoperative patients, and may reduce the incidence of postoperative pneumonia.

The study has some limitations. First, only a small number of patients were examined. Second, the actual occurrence of postoperative complications was not examined in the study. Future studies comprising a larger number of patients that includes investigation of the rate of postoperative complications will be necessary in order to address these shortcomings.

## Conclusions

Increased amount of tongue coating after surgery was associated with increased bacterial count in the saliva, and therefore tongue coating should be removed after surgery to minimize the risk for postoperative infectious complication in patients undergoing major oncologic or cardiac surgery.

## Abbreviation

PS: Performance status
